# Effect of Forced Convection on Magnesium Dendrite: Comparison between Constant and Altering Flow Fields

**DOI:** 10.3390/ma16247695

**Published:** 2023-12-18

**Authors:** Lang Qin, Ang Zhang, Jinglian Du, Zhihua Dong, Feng Liu, Bin Jiang

**Affiliations:** 1 National Engineering Research Center for Magnesium Alloys, National Key Laboratory of Advanced Casting Technologies, College of Materials Science and Engineering, Chongqing University, Chongqing 400044, Chinajiangbinrong@cqu.edu.cn (B.J.); 2 State Key Laboratory of Solidification Processing, Northwestern Polytechnical University, Xi’an 710072, Chinaliufeng@nwpu.edu.cn (F.L.)

**Keywords:** dendrite, solidification, magnesium, forced convection, phase-field simulation

## Abstract

Convection has a nonnegligible effect on the growth of the magnesium dendrite with six-primary-branch pattern. Most work, however, investigates the effect of the convection by simplifying the melt flow as a constant horizontal flow. In this work, four convection behaviors, including equally distributed convection, linearly distributed convection, sinusoidal-wave convection, and square-wave convection, are imposed and simulated through the phase-field lattice-Boltzmann schemes. The effects of constant (the former two) and altering (the latter two) flow fields are quantified by the length ratio of the upstream primary arm to the downstream one. The results show that the dendrite asymmetry increases under the constant forced convections but presents nonmonotonic change under the altering convections. A simple mathematical relation is fitted to summarize the dependence of the dendrite asymmetry on the input velocity, the undercooling, and the flow frequency. Deep understanding of the convection effects can guide the prediction and control of the magnesium dendrite under more complex situations.

## 1. Introduction

As the lightest metallic structural material, magnesium (Mg) alloy has attracted increasing attention with superior properties such as high specific strength, good damping performance, and large hydrogen storage capacity [1,2,3,4]. More than 90 percent of Mg products are manufactured by cast Mg alloys which are formed by smelting and solidification. During this process, the filling condition changes dramatically, which directly affects melt convection and thus evolution of solidification microstructures [5,6,7,8]. Exploring the relationship between the convection and the Mg alloy microstructures can guide optimization of the microstructures and thus improvement of the material properties.

Influenced by crystallographic orientation and anisotropic surface energy, the solidification microstructure of the primary Mg commonly presents snowflake pattern named dendrite (i.e., six-primary-branch pattern), and the included angles between neighboring primary arms are 60° under pure diffusion condition [9,10]. However, available studies, including experiments and simulations, mostly focus on the microstructure control of the Al alloys with four-fold symmetry structure. Due to the hexagonal close-packed structure in the Mg alloys, the added complexity in the morphology makes the prediction and control of the Mg alloy microstructures more challenging. The solute transport is on a similar length scale to the dendrite growth. By causing solute redistribution, the unavoidable melt convection under actual conditions has non-negligible effects on the Mg dendrite morphology.

The common opinion is that the growth of the upstream arms is accelerated while the downstream ones are inhibited under a single-direction convection [11,12]. However, the practical convection is rather complex and the question concerning how the Mg dendrite changes under more general convection conditions remains to be answered. Shevchenko et al. [13] reported the effect of the horizontal forced convection on the growth of the Ga-In alloy with lower melting point. But restricted by the difficulty of building proper apparatus and limited observation resolution, neither generating complex but controllable convection conditions nor obtaining detailed flow field distribution around the growing dendrites is trivial work, much less the Mg dendrite with higher oxidation tendency [14,15].

The development of numerical techniques makes it possible to make predictions and deepen understanding of physics behind crystal growth. There are mainly two numerical methods including explicit interface tracking and diffuse interface approach. The phase-field method (PFM), as a typical diffuse interface approach, is becoming a standard tool to simulate microstructure evolution with rigor physical foundation [16,17,18,19]. The solid and liquid phases are denoted by the phase-field variable which changes smoothly across the diffuse interface, and the phase transformation is characterized by the spatial-temporal evolution of the phase-field variable [20,21]. To solve the flow field in the dendrite skeleton, a kinetic based method named the lattice Boltzmann method (LBM) is employed due to higher numerical stability and easier boundary setting than the conventional Navier-Stokes solver [22,23]. Combining the PFM and the LBM to solve the liquid flow during dendrite growth has been tried by Medvedev et al. [24], Takaki et al. [25], and Zhang et al. [17].

In this work, to simulate more general convection conditions, four cases of convection settings, including equally distributed convection, linearly distributed convection, sinusoidal-wave convection, and square-wave convection, are investigated and compared. The convection magnitude can change with time and space. Despite being idealized, the simulated convection conditions can be thought as an abstraction of real complex flow conditions, e.g., adopting the linearly distributed flow field to simulate unequal flow velocities along the radial direction during stirring operation [26,27,28] and adopting altering flow fields (sinusoidal-wave convection and square-wave convection) to simulate reciprocating flow during vibration operation [29,30,31]. The change of the dendrite symmetry under different convections are quantified by measuring the arm length. The main objective is to gain new insight into the dendrite growth of the Mg alloys with the six-primary-branch pattern under the influence of complex convection fields.

## 2. Methods

### 2.1. Phase-Field Method

A phase-field variable *ϕ* is introduced to denote the solid and liquid phases. *ϕ* changes from 1 in solid to −1 in liquid across a numerically resolvable diffuse interface, which naturally distinguishes the two phases. The system evolution is determined by solving a stiff system of partial differential equations without explicit tracking of the macroscopic sharp interfaces. The solute diffusion field is extended by incorporating the liquid convection which is driven by the forced inlet velocity, and an antitrapping solute flux is considered to eliminate unphysical effects caused by unequal diffusivities in solids and liquids [32]. Derived from the free energy functional, the governing equations are expressed as [33,34,35]

(1) $$\begin{array}{c} A{\left(n\right)}^{2}\left[MC_{\infty }\left(1+\left(1-k\right)U\right)\right]\frac{\partial \phi }{\partial t}\\ \;=\frac{1}{2}\nabla \cdot \left[\frac{\partial (A{\left(n\right)}^{2}{\left|\nabla \phi \right|}^{2})}{\partial \nabla \phi }\right]+\phi \left(1-\phi^{2}\right)-\lambda {\left(1-\phi^{2}\right)}^{2}(\theta \\ \;+MC_{\infty }U) \end{array}$$

(2) $$\begin{array}{c} \frac{\left(1+k\right)-\left(1-k\right)\phi }{2}\frac{\partial U}{\partial t}\\ \;=\nabla \cdot \left[D\frac{1-\phi }{2}\nabla U+\frac{1+\left(1-k\right)U}{2\sqrt{2}}\frac{\partial \phi }{\partial t}\frac{\nabla \phi }{\left|\nabla \phi \right|}\right]\\ \;+\frac{1+\left(1-k\right)U}{2}\frac{\partial \phi }{\partial t}-\frac{\left(1-\phi \right)\left(1-k\right)}{4}v\\ \;\cdot \left\{\left[1+k-\left(1-k\right)\phi \right]\nabla U-\left[1+\left(1-k\right)U\right]\nabla \phi \right\} \end{array}$$

where *M*, *D*, *U*, and *θ* are the dimensionless liquidus slope, the dimensionless solute diffusivity, the dimensionless solute concentration and the dimensionless temperature,

*M* = |*m*|(1 − *k*)/∆*T*_0_ (3)

*D* = 0.6267*λ* (4)

(5) $$U=\frac{\frac{2C/C_{\infty }}{1+k-\left(1-k\right)\phi }-1}{1-k}$$

*θ* = (*T* − *T_M_ − mC*_∞_)/∆*T*_0_ (6)

where *m*, *k*, ∆*T*_0_, *λ*, *T_M_*, and *C_∞_* are the liquidus slope, the partition coefficient, the freezing range, the coupling constant, the melting point of pure Mg, and the initial solute concentration, respectively. ∆ = −*θ* is the undercooling.

The anisotropy function *A*(***n***) characterizing the six-primary-branch pattern of the Mg dendrite is expressed as

*A*(***n***) = 1 + *ε* cos(6(*ѱ* − *ѱ*_0_)) (7)

where ***n*** = −**∇***ϕ*/|**∇***ϕ*| is the unit vector normal to the solid-liquid interface, *ε* is the anisotropy strength, *ѱ* = arctan((*ǝϕ*/*ǝy*)/(*ǝϕ*/*ǝx*)) is the angle between the primary arm and the *x*-axis, and *ѱ*_0_ is the initial angle.

### 2.2. Lattice Boltzmann Method

The liquid flow is calculated by the LBM, in which a collection of particles is introduced to describe the macro liquid. Two repeated operations of the particles, streaming and collision, are performed to update the liquid flow, and attention is paid to the solution to the evolution of the particle distribution function [36,37,38,39]. Constructed on Cartesian lattices with diagonal links, the particle distribution function *f_i_* satisfies

(8) $$f_{i}\left(r+\delta r,\;t+\delta t\right)-f_{i\;}\left(r,\;t\right)=-\frac{1}{\tau_{f}}\left[f_{i}\left(r,\;t\right)-f_{i}^{eq}\left(r,t\right)\right]+\delta tF_{i}$$

where the superscript *eq* denotes the equilibrium state,

(9) $$f_{i}^{eq}=\rho w_{i}\left[1+\frac{3e_{i}\cdot v}{c^{2}}+\frac{9{\left(e_{i}\cdot v\right)}^{2}}{2c^{4}}-\frac{3v\cdot v}{2c^{2}}\right]$$

where *ρ* is the particle density, *τ_f_* is the relaxation time which is connected with the kinematic viscosity *υ_k_* by *υ_k_* = *c*^2^*δt*(2*τ_f_* − 1)/6, *c* = *δx*/*δt*, ***e_i_*** is the discrete velocity, *w_i_* is the corresponding weight coefficient, and *F_i_* is the discrete force exerted on the solid dendrite.

(10) $$F_{i}=\left(1-\frac{1}{2\tau_{f}}\right)w_{i}\left[\frac{3\left(e_{i}-v\right)}{c^{2}}+\frac{9(e_{i}\cdot v)e_{i}}{c^{4}}\right]\cdot F_{d}$$

(11) $$F_{d}=-\frac{2.757\rho \upsilon_{k}\left(1-\phi \right)\left(1+\phi^{2}\right)}{4W_{0}{}^{2}}v$$

where *W*_0_ is the measure of the interface width. ***F_d_*** acts as a momentum sink that drags the surrounding liquid and reproduces the no-slip boundary condition in the diffuse interface region.

Accordingly, the flow velocity is calculated as

(12) $$v=\sum_{i}f_{i}e_{i}/\rho +\delta tF_{d}/\left(2\rho \right)$$

After the flow velocity is updated and input into Equation (2), the convection-diffusion transport of the alloy solute is solved. The system evolution is updated through iteratively solving Equations (1), (12), and (2) in sequence.

### 2.3. Convection Conditions

**Figure 1:**
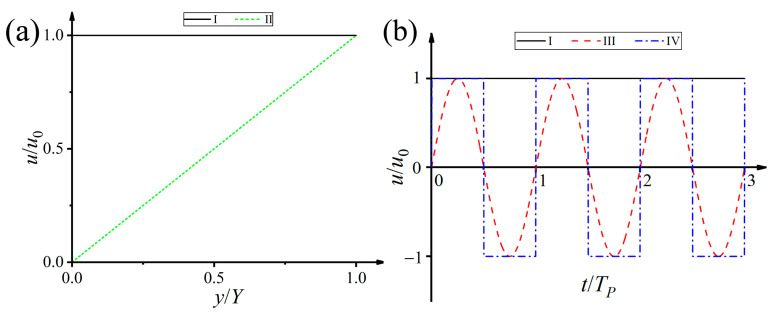
Four cases of convection conditions. *u*_0_ is the given horizontal flow velocity, and *T_p_* = 2*π*/*ω* is the period of the periodic flow field. (**a**) Cases I and II. (**b**) Cases I, III, and IV.

Four cases of convection conditions, as shown in Figure 1, are imposed by assigning the input velocities with different values. The expressions of the inlet velocity vector ***v*** = (*u*, *v*) in the four cases are expressed as

(13) $$\begin{array}{c} I:\;u=u_{0}\\ \mathrm{II}:\;\;u=u_{0}y/Y\\ \mathrm{III}:\;u=u_{0}sin\omega t\\ \mathrm{IV}:\;u=u_{0}{\left(-1\right)}^{floor\left(\omega t/\pi \right)} \end{array}$$

where *u*_0_ is the given horizontal flow velocity component, and *v* = 0 is the longitudinal velocity component. The operator *floor* denotes the rounding down operation, *ω* = 2*π*/*T_p_* is the frequency of the periodic flow field, and *T_p_* is the period. Different from the convectional convection behavior (Case I), both the magnitude and the direction of the liquid flow can change with time in Cases II and III. In Case IV, the input velocity changes with the *y* coordinate, i.e., nonequal flow velocity perpendicular to the flow direction.

### 2.4. Computation Settings

The dendrite growth of the Mg-6 wt.%Gd alloy is simulated. The Mg-Gd alloys, as one of the widely used Mg-rare earth alloys, have excellent mechanical properties and good heat resistance [4,40,41]. Due to the large phase contrast under the exposure of collimating X-ray, the solidification behavior of the Mg-6 wt.%Gd alloy can be in situ observed by X-ray synchrotron radiography, which provides experimental references for the present simulations.

The simulation parameters are set as follows. The domain size *X* × *Y* is 1024 × 1024 in mesh units, and the seed radius is 4 in mesh units, with the real mesh size *dx* of 4.34 × 10^−8^ m, i.e., 0.8 *W*_0_, where 0.8 *W*_0_ is the interface thickness. The time step *dt* is 2.04 × 10^−2^ *τ*_0_, i.e., 1.26 × 10^−7^ s. Until defined otherwise, the dimensionless undercooling is 0.20, the anisotropy strength is 0.02, the initial orientation angle is 0°, the flow field frequency is 0.10, and the dimensionless inlet velocity is 0.05. A frozen temperature approximation is set inside the domain.

The liquid flows from left to right, i.e., an assigned inlet velocity is set at the left side and a zero-velocity gradient boundary is set at the right side. At the top and bottom sides, no slip boundary conditions are set for the flow field. At the solid-liquid interface, the liquid fraction of 0.10 is set as the boundary satisfying the bounce-back velocity scheme in the LBM, i.e., vanishing the liquid flow velocity here and updating the flow according to the bounce-back principle [22]. For the phase field and solute field, zero-Neumann boundary conditions are set at all the walls.

The mesh layout covering the dendrite tip is illustrated in [42], in which the number of the meshes across the diffuse interface (*ϕ* changing from −1 in liquid to 1 in solid) is set to 16. The grid sensitivity is tested in terms of the tip growth velocity. When the dimensionless mesh size *dx*/*W*_0_ reduces from 3.2 to 0.4, the growth velocity increases but the increase amplitude decreases. The velocity difference between *dx*/*W*_0_ = 0.8 and *dx*/*W*_0_ = 0.4 is less than 4%. Too small of a mesh size causes enormous computing overhead, while too large of a size will deteriorate numerical accuracy. To balance the efficiency and the accuracy, *dx*/*W*_0_ = 0.8 is fixed.

The simulations are run on a parallel platform based on message passing interface. The number of the discrete meshes is over one million, and the average elapsed time is about 1200 s for a total of 10,000 steps when eight processes are parallelized. The phase-field lattice-Boltzmann method has been validated from dendrite growth and fluid dynamics in our recent work [17].

## 3. Results

**Figure 2:**
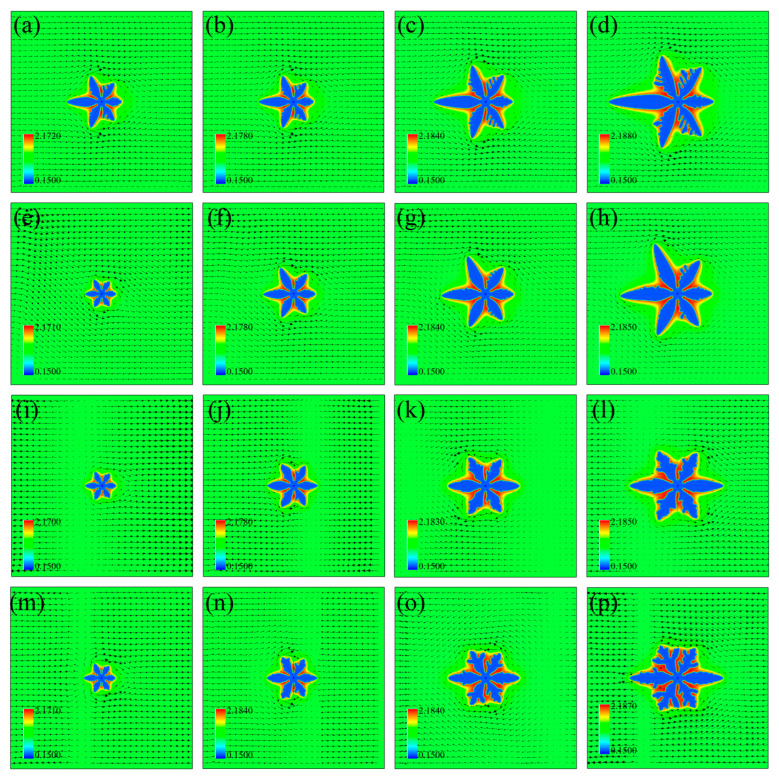
Evolution of Mg dendrite under the four convection behaviors. Each column corresponds to the time *t*_1_–*t*_4_ from left to right with *t*_1_/t^*^ = 2, *t*_2_/t^*^ = 4, *t*_3_/t^*^ = 6, *t*_4_/t^*^ = 8 and t^*^ = 1000 *dt*. The arrows denote the flow velocity vectors. (**a**–**d**) Solute field distribution in Case I. (**e**–**h**) Solute field distribution in Case II. (**i**–**l**) Solute field distribution in III. (**m**–**p**) Solute field distribution in Case IV. The frequency of the flow field is *ω* = 0.1, the dimensionless undercooling is ∆ = 0.2, and the inlet velocity is *u*_0_ = 0.05.

Figure 2 shows the evolution of the simulated Mg dendrite under the four convection conditions. The arrows, superimposed on the solute field cloud map, denote the flow velocity vectors. The denser the arrows, the larger the flow velocity. The Mg dendrite presents a typical six-primary-branch pattern by stretching six arms with the included angle of 60° from the seed center. The rejected solute enriches at the interdendritic root, forming a nonuniform solute boundary layer surrounding the dendrite.

The dendrite morphology presents remarkable difference between each other due to difference of the four convection behaviors. In Case I, the from-left-to-right melt bypasses the solid dendrite by crossing the dendrite tip and converges at the downstream side. The solute is transported towards the downstream side and enriches there, which reduces the local undercooling (i.e., driving force) and slows down the downstream dendrite growth. The upstream dendrite length is much longer than the downstream one due to difference of the growth driving force. A typical asymmetric dendrite pattern is observed and such asymmetry increases with time.

The difference between Case I and Case II is that the input velocity is non-constant in space and that between Case I and Case III (or Case IV) is that the velocity is independent of time. The input velocity increases linearly along the *y* axis, and the melt flows from the upper-left to the lower-right parts, as shown in Figure 2e. The stronger convection at the upper half accelerates more solute transporting along the upper dendrite boundary, causing more developed upper half but underdeveloped lower half of the solid dendrite. Similar to the first row in Figure 2, the melt reaches the downstream side through bypassing the dendrite, and more enriched solute inhibits the downstream growth.

In Cases III and IV, the altering flow field weakens the dendrite asymmetry since the rejected solute is not necessarily accumulated at a fixed side. The sidebranches can emerge at both sides of the primary arms, as shown in Figure 2l,p, which is different from the sidebranches only generating at the upstream side in the first two rows. The direction of the input inlet velocity changes with time, causing a zero-velocity region inside the domain. Taking Figure 2j for instance, the input melt flows from left to right, while that in the last period is from right to left. The two opposite convections impinge with each other in front of the right primary arm, causing a zero-velocity region, similarly for those in the other moments in the last two rows. The zero-velocity region is equivalent to the origin (e.g., Figure 2i) or the end (e.g., Figure 2j) of two opposite flows, which is considered as a unique feature of such periodic flow with changing directions.

**Figure 3:**
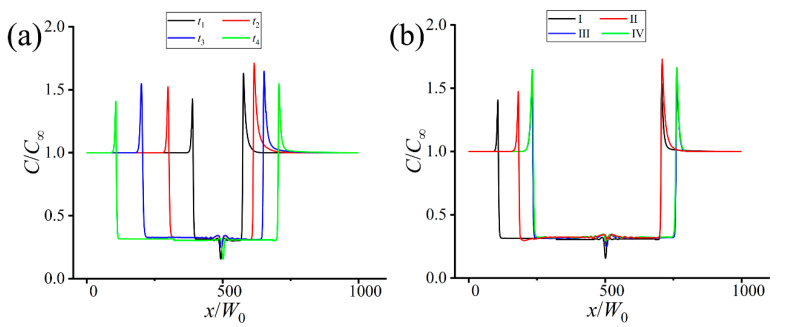
Solute concentration vs the distance along the domain horizontal centerline in Figure 2. (**a**) The first row in Figure 2. (**b**) The last column in Figure 2.

Figure 3 shows the change of the solute concentration along the domain horizontal centerline. The solute concentration reaches the extreme at the solid-liquid interface, and the distance between the upstream and downstream extreme points denotes the width of the solid dendrite. In Figure 3a, the extreme concentration changes nonmonotonically as the dendrite grows, i.e., the upstream one first increases from *C*/*C*_∞_ = 1.428 (*x*/*W*_0_ = 383.743) at *t*_1_ to *C*/*C*_∞_ = 1.549 (*x*/*W*_0_ = 197.096) at *t*_3_ and then decreases to *C*/*C*_∞_ = 1.408 (*x*/*W*_0_ = 106.431) at *t*_4_. Such change can be attributed to the competition between solute trapping and convection transport during dendrite growth. Despite nonmonotonic change of the concentration extreme, the downstream concentration extreme is always higher than the upstream one due to solute enrichment at the downstream side.

The change of the solute concentration in the four cases in Figure 3b illustrates the difference caused by the convection behaviors. Both the upstream concentration extreme and its position in Case I are minimum, indicating the horizontal upstream dendrite arm is the longest in Case I. The length of the horizontal upstream primary dendrite arm in Case II is between Case I and Case III (or Case IV), while that of the horizontal downstream arm is the same as Case I, albeit higher concentration extreme in Case II. The concentration-distance curves in Cases III and IV overlap, corresponding to the similar solute field cloud maps in Figure 2l,p. The concentration extremes at both the upstream and downstream sides are also similar under the two periodic flow fields.

**Figure 4:**
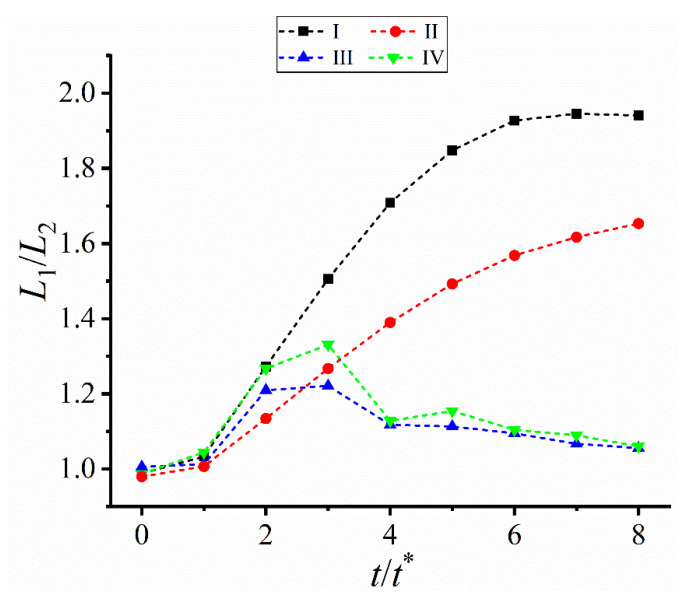
Length ratio *L*_1_/*L*_2_ of the horizontal primary arms vs time *t*/t^*^. *L*_1_ and *L*_2_ are the length of the upstream and downstream dendrite arms respectively. t^*^ = 1000 *dt*.

The above results further prove the dendrite asymmetry under the forced convection. To characterize such asymmetry, the change of the length ratio of the horizontal upstream primary arm *L*_1_ to the downstream one *L*_2_ is measured, as shown in Figure 4. *L*_1_/*L*_2_ in Cases I and II keeps increasing, while a nonmonotonic change is found in Cases III and IV. This difference indicates a strong dependence of the dendrite morphology on the imposed flow field. Due to the larger average inlet velocity, *L*_1_/*L*_2_ in Cases I is always larger than that under II, similarly for Case IV larger than Case III. The nonmonotonic change in Cases III and IV, together with local fluctuation, is attributed to the change of the flow direction, which weakens one-side concentration accumulation and thus narrows the upstream-downstream difference. But *L*_1_/*L*_2_ in the four cases are all larger than one due to more solute accumulated in the left domain side.

## 4. Discussion

The four convection cases can be grouped into two categories including constant (Cases I and II) and altering flow fields (cases III and IV). The input velocity of the constant flow fields is independent of time but the convection strength can change (i.e., Case II), while the altering flow fields involve simultaneous change of both convection strength and convection direction. How the dendrite morphology, especially the dendrite symmetry, evolves in the four cases is discussed and compared.

### 4.1. Effect of Constant Flow Fields

**Figure 5:**
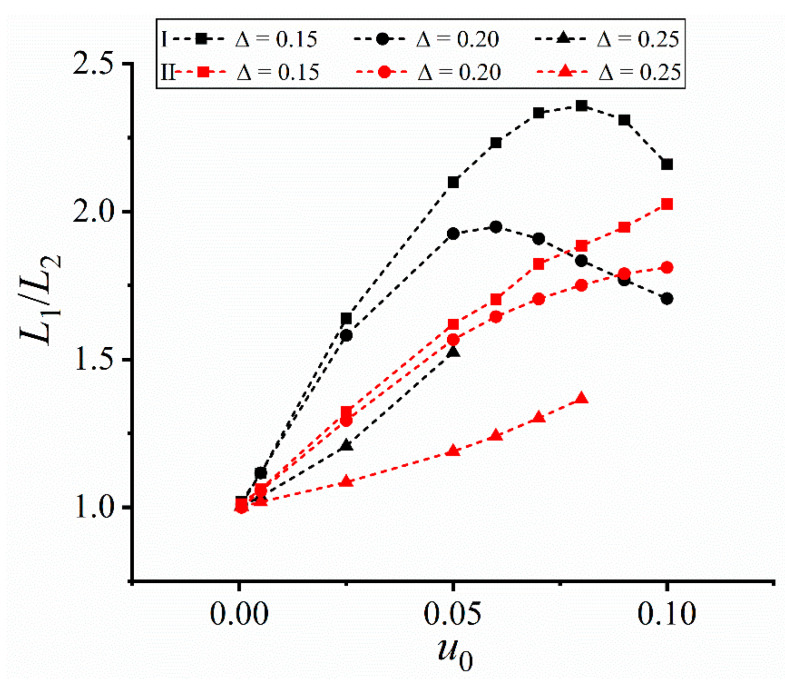
Length ratio *L*_1_/*L*_2_ of the horizontal primary arms vs the inlet velocity *u*_0_ in Cases I and II.

Figure 5 shows the change of the length ratio *L*_1_/*L*_2_ vs. the input velocity *u*_0_. *L*_1_/*L*_2_ > 1 in Cases I and II, meaning that the length of the upstream dendrite arm is always larger than that of the downstream one under the constant unidirectional flow field. The upstream arms are more developed and the dendrite asymmetry increases with the inlet velocity.

In Case I, *L*_1_/*L*_2_ first increases and then decreases at ∆ ≤ 0.20, and such nonmonotonic change is attributed to the blocking effect of the left domain wall. The left horizontal primary arm grows so fast under larger input velocities that being blocked by the left domain wall, while the right one is less affected due to shorter length. The limited increase of the left primary arm causes the decrease of *L*_1_/*L*_2_, and the larger the undercooling, the more significant the blocking effect, i.e., showing the turning point earlier. Thus, the input velocity corresponding to the *L*_1_/*L*_2_ extreme at ∆ = 0.20 is less than that at ∆ = 0.15. When the undercooling reaches 0.25, the left horizontal primary arm reaches the left wall at *u*_0_ > 0.05, making *L*_1_/*L*_2_ not measurable.

In Case II, the left horizontal primary arm does not impinge the domain wall due to weaker flow intensity than that in Case I. *L*_1_/*L*_2_ keeps increasing and the values are lower than those in Case I.

The effect of the orientation angle *ѱ*_0_ is also evaluated by changing *ѱ*_0_ from 0° to 30°. The location where the melt flows towards changes from the dendrite tip at *ѱ*_0_ = 0° to the interdendrite root at *ѱ*_0_ > 0°. Similarly, the upstream dendrite arms at the left half of the domain are always more developed, and the primary arm with a smaller included angle with the *x* axis is more sensitive to the change of the flow condition. The included angle at the upstream side increases while that at the downstream decreases, accompanied by the impingement of developed sidebranches in the interdendrite root.

### 4.2. Effect of Altering Flow Fields

**Figure 6:**
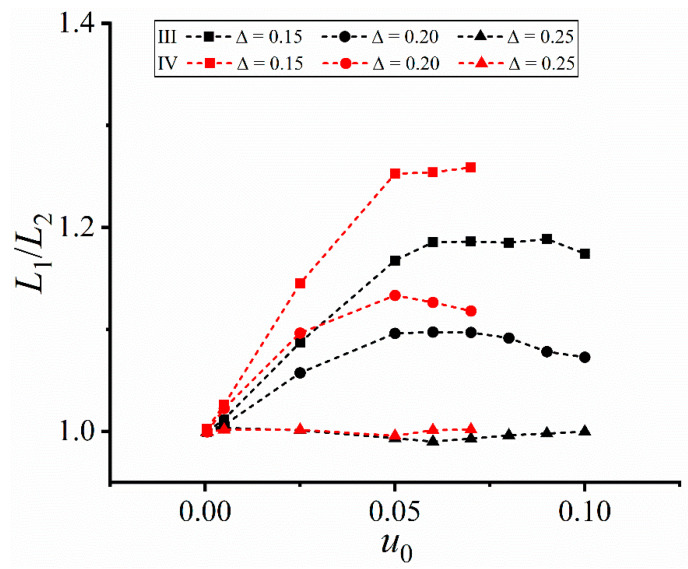
Length ratio *L*_1_/*L*_2_ of the horizontal primary arms vs the inlet velocity *u*_0_ in Cases III and IV. The frequency of the flow field is *ω* = 0.10.

Figure 6 shows the change of *L*_1_/*L*_2_ under the two altering flow fields. The values of *L*_1_/*L*_2_ are relatively small compared with those in Figure 5. At early solidification, a large amount of solute is transported towards downstream due to less resistance surrounding the smaller dendrite. The greater the input velocity, the stronger the transport capacity, and thus the more significant the dendrite asymmetry. When the direction of the flow field changes, the solute is transported towards the opposite side. But both the resistance and the moving path are larger than those at the last time, which weakens the dendrite asymmetry but cannot reverse such asymmetry. The reciprocating flow occurs at the next time when the flow direction changes. Accordingly, the values of *L*_1_/*L*_2_ under the altering flow fields are smaller than those in Figure 5.

*L*_1_/*L*_2_ remains at a relatively stable value after imposing larger input velocities in Cases III and IV, e.g., *u*_0_ ≥ 0.05. The stable stage results from the complex interaction between the forced convection and the undercooling, i.e., the competition between convection transport and solute trapping. As a result of a limited growth capacity at a given undercooling, the effect of increasing input velocity on the upstream-downstream arm difference diminishes, i.e., the solute trapping capacity can restrict the change of the convection-induced asymmetry. Besides, a larger undercooling corresponds to a stronger solute trapping, i.e., less affected by the convection transport, and thus *L*_1_/*L*_2_ decreases with the increase of the undercooling, which agrees with those in Figure 5.

**Figure 7:**
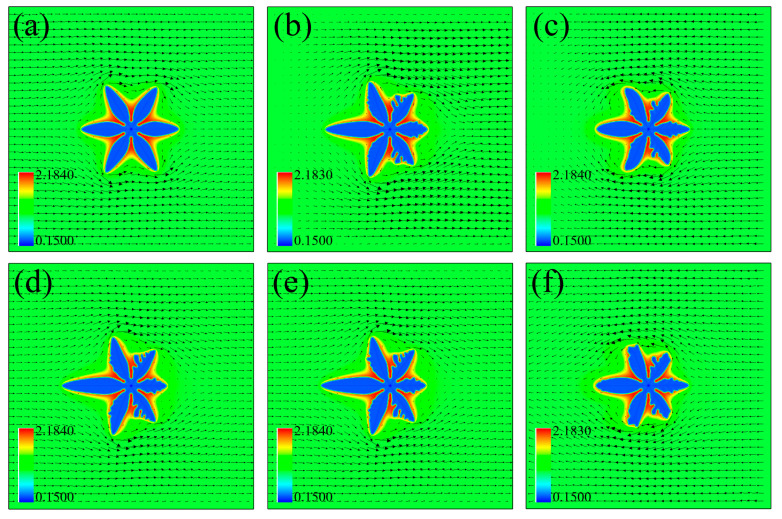
Typical Mg dendrite under different flow frequencies. The arrows denote the flow velocity vectors. (**a**–**c**) Solute field distribution in Case III. (**d**–**f**) Solute field distribution in Case IV. The frequencies of the flow field are *ω* = 0.001, 0.025, and 0.05 from left to right. The dimensionless undercooling is ∆ = 0.2, and the inlet velocity is *u*_0_ = 0.05.

When the frequencies of the altering flow fields change, a nonmonotonic change is observed, as shown in Figure 7. The length of the left horizontal primary arm is longer than that of the right one, but such difference changes with the flow frequency. The largest upstream-downstream arm difference in Figure 7 is in the center column, indicating a nonnegligible effect of flow frequency on the dendrite growth dynamics.

**Figure 8:**
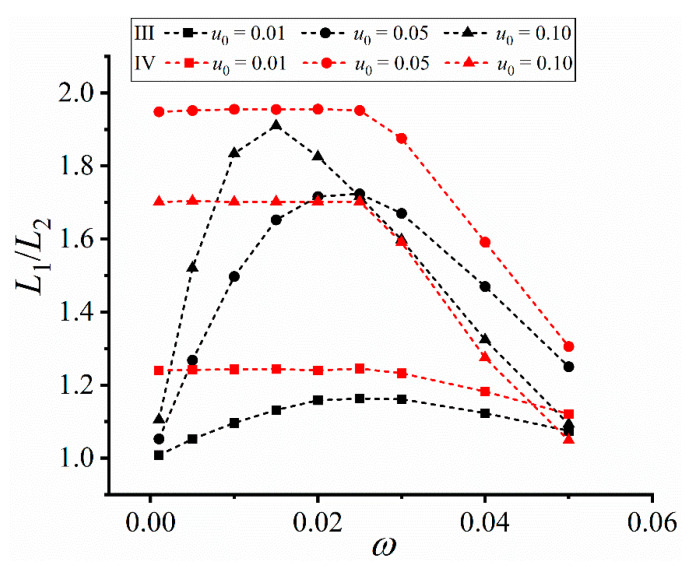
Length ratio *L*_1_/*L*_2_ of the horizontal primary arms vs the frequency *ω* in Cases III and IV. The dimensionless undercooling is ∆ = 0.2.

Figure 8 shows the change of the length ratio *L*_1_/*L*_2_ vs the flow frequency *ω*. In Case III, an analogous parabolic relation between *L*_1_/*L*_2_ and *ω* is established, and the coordinates of the extreme points are (0.025, 1.163), (0.025, 1.724), and (0.15, 1.910) for *u*_0_ = 0.01, 0.05, and 0.10, respectively. Too high or too low frequencies both correspond to lower *L*_1_/*L*_2_, indicating that the altering behavior of the sinusoidal-wave flow field causes asymmetry in a medium range. Similar effect can also be obtained at high frequencies in Case IV, where *L*_1_/*L*_2_ decreases with the increase of *ω* at *ω* > 0.025, decreasing asymmetry. Differently, the low frequencies in Case IV generate a relatively stable *L*_1_/*L*_2_, indicating the square-wave convection with large periods is somewhat equivalent to the unidirectional flow case (i.e., independent of the flow frequency). It is noted that the maximum input velocity does not necessarily correspond to the maximum *L*_1_/*L*_2_, e.g., the maximum *L*_1_/*L*_2_ at *u*_0_ = 0.10 is less than that at *u*_0_ = 0.05 in Case IV. Such results further indicate the complex competition between convection (i.e., convection transport) and undercooling (i.e., solute trapping).

### 4.3. Parameter Evaluation

The evolution of the Mg dendrite under forced convection is affected by the combined effects of the undercooling and the flow characteristics (e.g., the input velocity and the flow frequency). To evaluate the dependence of the morphological features on the growth conditions, a thorough fitting analysis is performed based on the above simulation results. Since the change of *L*_1_/*L*_2_ presents linear and analogous parabolic relations with variables of interest, a combination of multiple polynomial functions is adopted, i.e.,

(14) $$L_{1}/L_{2}=1+k_{1}u_{0}\Delta \left(1+k_{2}u_{0}{}^{2}\right)\left(1+k_{3}\Delta^{2}\right)\left(1+k_{4}\omega +k_{5}\omega^{2}+k_{6}\omega^{3}\right)$$

where *k_i_* (*i* = 1–6) is the coefficient.

In Cases I and II, the flow field is independent of time, and the flow frequency is zero. Equation (14) is recast to

(15) $$L_{1}/L_{2}=1+k_{1}u_{0}\Delta \left(1+k_{2}u_{0}{}^{2}\right)\left(1+k_{3}\Delta^{2}\right)$$

Table 1 lists the fitting values of the coefficients in Equations (14) and (15), in which reduced chi-square and adjusted R-square are two indicators measuring the nonlinear fitting degree. Reduced chi-square is equivalent to mean square of residual and the closer to 0, the better the fitting degree. For adjusted R-square, a value closer to 1 means a higher fitting degree. The values of the two parameters in Table 1 clearly show the reasonability of the nonlinear fitting.

If focusing on a certain variable, e.g., the input velocity or the flow frequency, Equations (14) and (15) can be further recast to simpler forms by assigning the values of the other variables. Taking the input velocity for instance, the fitting functions at ∆ = 0.15 are

(16) $$\begin{array}{c} function:\;L_{1}/L_{2}=1+k_{1}u_{0}\left(1+k_{2}u_{0}{}^{2}\right)\\ Case\;I:\;L_{1}/L_{2}=1+0.2u_{0}\left(1-0.003u_{0}{}^{2}\right)\\ Case\;\mathrm{II}:\;L_{1}/L_{2}=1+0.13u_{0}\left(1-0.02u_{0}{}^{2}\right)\\ Case\;\mathrm{III}:\;L_{1}/L_{2}=1+0.373u_{0}\left(1-0.0057u_{0}{}^{2}\right)\\ Case\;\mathrm{IV}:\;L_{1}/L_{2}=1+0.047u_{0}\left(1-0.0003u_{0}{}^{2}\right) \end{array}$$

where the functions for Cases I and II correspond to Figure 5 and those for Cases III and IV are for Figure 6. The largest reduced chi-square is 0.0373 and the smallest adjusted R-square is 0.829, indicating the reasonability of the numerical fitting.

For the flow frequency in Figure 8, the undercooling is fixed at 0.2, and the fitting functions of the six curves with respective input velocities are

(17) $$\begin{array}{c} function:\;L_{1}/L_{2}=1+k_{1}\left(1+k_{4}\omega +k_{5}\omega^{2}+k_{6}\omega^{3}\right)\\ Case\;\mathrm{III}:\\ \{\begin{array}{c} u_{0}=0.01:\;L_{1}/L_{2}=1-0.040\times \left(1-1.149\omega -0.0414\omega^{2}+0.0149\omega^{3}\right)\\ u_{0}=0.05:\;L_{1}/L_{2}=1-0.240\times \left(1-1.250\omega +0.0625\omega^{2}+0.00575\omega^{3}\right)\\ u_{0}=0.10:\;L_{1}/L_{2}=1-0.554\times \left(1-1.408\omega +0.225\omega^{2}-0.010\omega^{3}\right) \end{array}\\ Case\;\mathrm{IV}:\\ \{\begin{array}{c} u_{0}=0.01:\;L_{1}/L_{2}=1+0.228\times \left(1+0.00167\omega +0.0150\omega^{2}-0.00230\omega^{3}\right)\\ u_{0}=0.05:\;L_{1}/L_{2}=1+0.900\times \left(1+0.00111\omega +0.0178\omega^{2}-0.00288\omega^{3}\right)\\ u_{0}=0.10:\;L_{1}/L_{2}=1+0.658\times \left(1+0.000714\omega +0.0229\omega^{2}-0.00386\omega^{3}\right) \end{array} \end{array}$$

with the largest reduced chi-square and the smallest adjusted R-square being 0.00243 and 0.958 respectively. A high fitting is established though the unchanged horizontal stage at low frequencies in Case IV.

Although higher fitting degree is obtained according to Table 1, Equations (14) and (15) are actually mathematical statistical results with less sound physics. More rigorous analysis could be obtained by machine learning in which large amounts of testing data are trained based on physical-informed models. The present work can be considered as a preliminary attempt to explore the effects of complex convection transports. Combining the present work with experiments and/or physical-informed algorithms will be more appealing in establishing a more unified prediction model.

## 5. Conclusions

The growth of the magnesium dendrite under four convection conditions is simulated and compared through the phase-field lattice-Boltzmann method. The forced convection is driven by imposing different input velocities at the left domain wall, which can be classified into two kinds including constant (equally distributed convection and linearly distributed convection) and altering flow fields (sinusoidal-wave convection and square-wave convection). The change of the dendrite morphology is quantified by measuring the length ratio *L*_1_/*L*_2_ of two opposite growing primary arms along the flow direction. The magnesium dendrite grows under the complex interaction between the forced convection and the undercooling (i.e., the competition between convection transport and solute trapping). Through detailed comparison of the four convection cases, the following conclusions can be drawn.

(1) Under the constant flow fields, the dendrite becomes asymmetrical and the downstream dendrite arms are less developed due to more accumulated solute. If the input velocity changes linearly along the *y* axis, the melt will flow from the upper-left to the lower-right parts, and the stronger convection at the upper half will generate more developed upper half but underdeveloped lower half of the solid dendrite. The length ratio *L*_1_/*L*_2_ increases with the inlet velocity, but a nonmonotonic change can occur due to blocking effect of the domain wall. The larger the undercooling, the less the effect of the convection transport, and thus *L*_1_/*L*_2_ decreases with the increase of the undercooling.

(2) Under the altering flow fields, the dendrite asymmetry is less significant than that under the constant flow fields due to weakened one-side concentration accumulation under reciprocating flow, i.e., smaller *L*_1_/*L*_2_. A zero-velocity region is proved due to the impingement of two opposite convections inside the domain. *L*_1_/*L*_2_ presents a nonmonotonic relation with the variables of interest, e.g., input velocity and flow frequency. After imposing larger input velocities, *L*_1_/*L*_2_ remains at a relatively stable value, indicating that the solute trapping capacity can restrict the change of the convection-induced asymmetry. Besides, the maximum input velocity does not necessarily correspond to the maximum *L*_1_/*L*_2_, and the maximum asymmetry generally occurs in a medium frequency range.

(3) A fitting function, combination of multiple polynomial functions, is proposed to summarize the effect of the growth conditions including the input velocity, the undercooling, and the flow frequency. Albeit a mathematical statistics result, the proposed fitting functions is a preliminary attempt to quantify the effects of complex convection transports. More rigorous derivation can be extended through data training based on physical-informed algorithms.

## Figures and Tables

**Table 1:** Fitting values of the coefficients in Equations (14) and (15).

Case	*k*_1_	*k*_2_	*k*_3_	*k*_4_	*k*_5_	*k*_6_	Reduced Chi-Square	Adjusted r-Square
I	255	−60	−14.5	-	-	-	0.0123	0.945
II	125	−24	−13.5	-	-	-	5.389 × 10^−4^	0.995
III	24.7	−48	−15	769.231	−23,076.92	153,846.15	0.00414	0.944
IV	616	−70	−4.13	22.727	−1136.36	8227.27	0.00220	0.981

## Data Availability

Data will be made available on request.
